# A Radiation Oncologist’s Journey Through Technological Advancements in Oncology: Reflections on the Proton Therapy Winterschool at Paul Scherrer Institute, Switzerland

**DOI:** 10.7759/cureus.39551

**Published:** 2023-05-26

**Authors:** Nandan M Shanbhag

**Affiliations:** 1 Oncology/Palliative Care, Tawam Hospital, Al Ain, ARE; 2 Oncology/Radiation Oncolgy, Tawam Hospital, Al Ain, ARE

**Keywords:** rbe, advanced radiotherapy, precision radiotherapy, psi, proton therapy

## Abstract

The proton therapy course at the Paul Scherrer Institute (PSI) in Switzerland provided a comprehensive insight into the clinical, physics, and technological aspects of proton therapy, with a particular focus on pencil beam scanning techniques. The program consisted of engaging lectures, hands-on workshops, and facility tours, which covered topics such as the history of proton therapy, treatment planning systems, clinical applications, and future developments. Participants gained practical experience with treatment planning and simulation, while also exploring the challenges associated with various tumor types and motion management. The collaborative and supportive learning environment fostered by the faculty and staff at PSI enriched the educational experience, empowering participants to better serve their patients in the field of radiation oncology.

## Editorial

"The advantage of Protons is that they stop, the disadvantage of protons is that we don’t always know where...” - Prof. Tony Lomax, Paul Scherrer Institute, Switzerland

Prior to beginning my journey in radiation oncology, I was fascinated by the intersection of medical science and technology, which led me to a career focused on understanding and applying the latest advancements in cancer treatment. My extensive training and exposure to various cancer cases strengthened my determination to become an expert in radiation oncology. I developed a keen interest in cutting-edge radiation therapy techniques and sought to explore new frontiers in cancer treatment.

**Figure 1:**
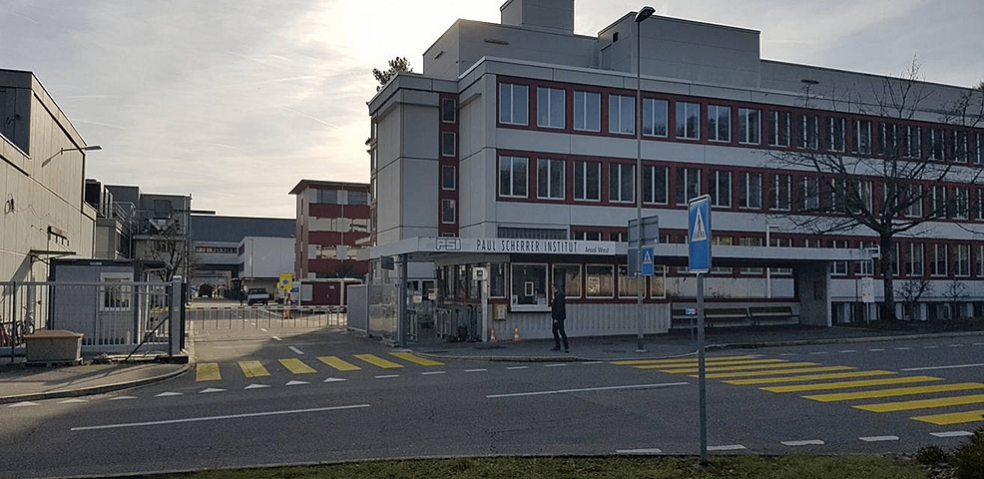
Paul Scherer Institute, Switzerland

My pursuit led me to the esteemed Paul Scherrer Institute (PSI) in Switzerland, a pioneer in proton therapy, where I enrolled in a comprehensive course on the clinical, physics, and technological aspects of proton therapy, particularly pencil beam scanning techniques (Figure 1).

The course, designed for professionals in radiation oncology and related fields, took place from January 22nd to January 25th, 2018 [1].

I arrived a day early at the beautiful Park Hotel - Bad Zurzach and was warmly greeted by Ms. Sibylle Bollhalder, who provided excellent support throughout the course. The well-organized course manual, containing color printouts of all presentations, served as a valuable resource during the program.

Day 1

The first day began with an introductory session by Professor Damien Weber, the Head of the Center for Proton Therapy at PSI. His warm welcome set the tone for the course, emphasizing the collaborative and interactive nature of the program. This was followed by a series of informative lectures on topics such as the history of proton therapy, treatment planning systems, and the clinical aspects of proton therapy.

**Figure 2:**
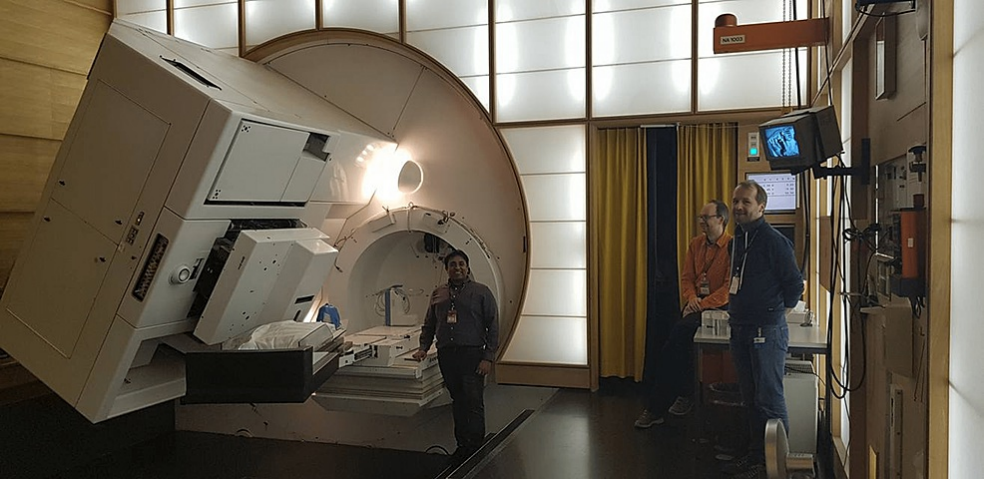
Gantry 1

The highlight of the day was a visit to the Gantry 1 facility, where the world's first proton gantry was installed. More than 1,300 patients were successfully treated at Gantry 1 and operations ceased at the end of 2018 (Figure 2).

We were given a guided tour of the facility by Dr Antony Lomax, a leading expert in proton therapy. The opportunity to see this groundbreaking technology up close was truly inspiring.

Day 2

On the second day, the lectures focused on the technical aspects of proton therapy, including beam delivery systems, imaging techniques, and quality assurance. Additionally, we had the opportunity to participate in hands-on workshops that allowed us to gain practical experience with treatment planning systems and the process of simulating patient treatment.

In the evening, the course participants were treated to a lovely dinner at a local restaurant, providing a relaxed setting to network and discuss the day's topics further.

Day 3

Day three delved deeper into the clinical applications of proton therapy, with presentations on pediatric cases, skull base tumors, and prostate cancer. These lectures not only showcased the latest advancements in proton therapy but also highlighted the importance of interdisciplinary collaboration in the management of complex cases.

**Figure 3:**
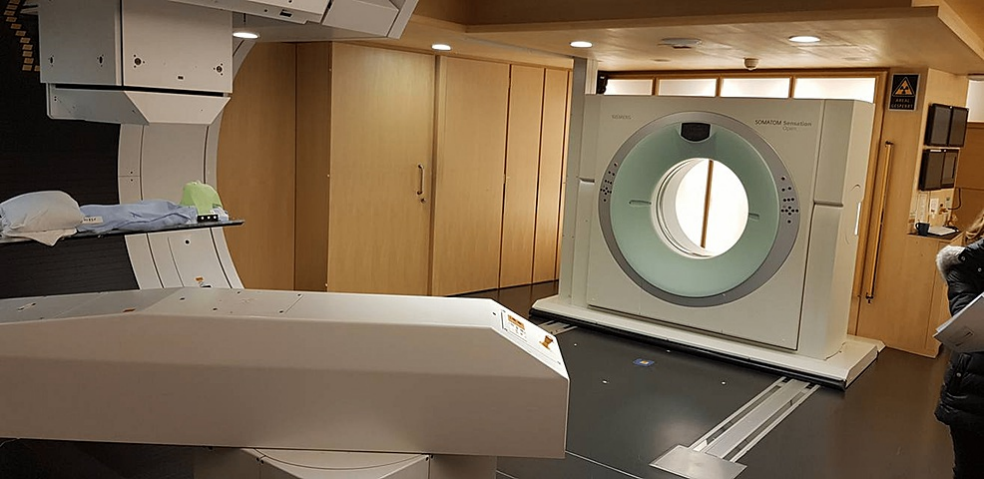
Gantry 2 with Computed Tomography on rails

**Figure 4:**
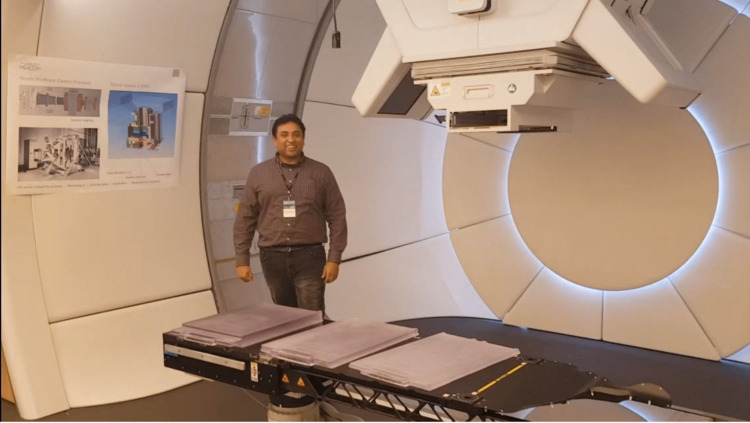
Gantry 3

**Figure 5:**
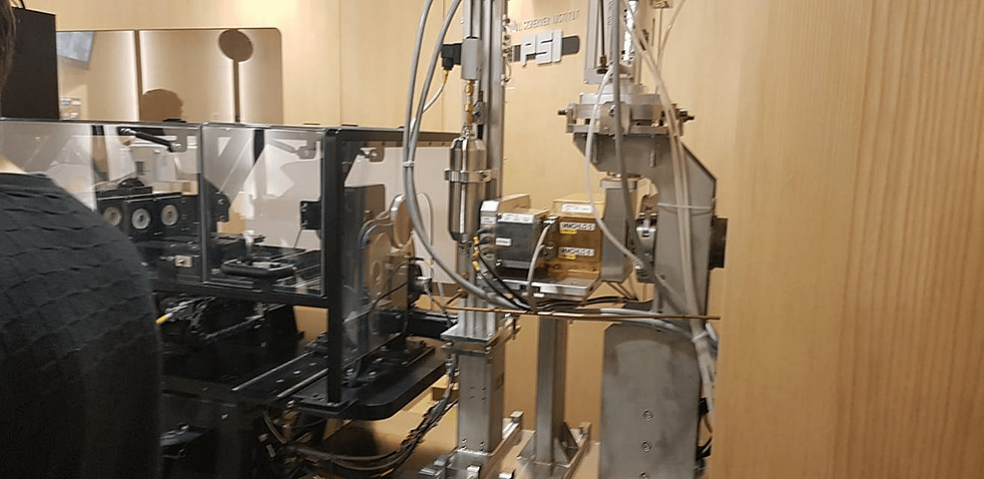
OPTIS 2 for treating ophthalmic tumors

The afternoon session featured a tour of Gantry 2 (Figure 3) and Gantry 3 (Figure 4) facilities, where we observed ongoing treatments and interacted with dedicated and knowledgeable staff. Since 1984, PSI has operated an ocular proton therapy program (OPTIS 2) for treating eye tumors, specifically ocular melanomas (Figure 5).

Day 4

On the final day of the course, we explored the future of proton therapy, including research and development efforts, and the potential role of artificial intelligence in treatment planning. We also had the chance to present and discuss our own clinical cases, fostering a collaborative and supportive learning environment.

The course concluded with a farewell ceremony, during which we received our certificates of completion.

Highlights of each day of the proton therapy course

On Day 1 of the proton therapy course at PSI, the day began with Prof. Damien Charles Weber's welcome introduction, which provided a brief history of radiation and proton therapy. The discussion moved on to the earliest use of radiation in eradicating parasites believed to cause cancer. Prof. Robert R. Wilson, a physicist, philosopher, and artist, was highlighted for his proposal of using fast protons in radiology. Prof. Wilson was the first to recognize the conformal nature of proton beams compared to photons, which allowed for higher doses to the target without increasing normal tissue exposure [2]. He acknowledged the learning curve associated with proton therapy and the lack of level I evidence despite treating over 13,000 patients around the world. The interplay effect, a patient-specific phenomenon, was also discussed. The interplay effect in proton therapy refers to the complex interplay between the motion of the patient's internal anatomy (e.g., respiratory motion) and the delivery of pencil beam scanning (PBS) proton therapy. This phenomenon can potentially impact the accuracy and effectiveness of the treatment.

In pencil beam scanning proton therapy, the treatment is delivered by scanning a narrow proton beam across the target volume, layer by layer. This is done by varying the energy and position of the beam during treatment. When the target volume, such as a tumor, is moving due to internal body motion (e.g., respiration), the proton beam and the moving target may not be perfectly synchronized. This can lead to an unintended dose distribution, with some areas receiving a higher or lower dose than intended.

The interplay effect is particularly relevant for tumors in regions prone to substantial motion, such as the thorax or abdomen [3]. The interplay effect is not the same as systematic errors in Photon therapy. Interplay effect refers to the variabilities that can occur in a patient due to the interaction between internal organ motion (e.g., respiratory motion) and the delivery of pencil beam scanning proton therapy. Systematic errors, on the other hand, are consistent deviations in treatment planning or delivery that can lead to inaccuracies in dose distribution.

The second lecture delved into the fundamental physics of proton therapy, highlighting the interactions of protons with atoms and their implications for precision and effectiveness. Scattering and energy loss were also discussed, as well as the choice of materials used in proton therapy equipment [4]. The interaction of protons with atoms is governed by three primary mechanisms: ionization, nuclear Coulombic interaction, and nuclear reactions. These interactions have profound implications for proton therapy's precision and effectiveness.

As protons penetrate tissue, they experience scattering and energy loss, with denser materials causing more scattering. Scattering is inversely proportional to atomic mass and directly proportional to atomic number, a crucial consideration in proton therapy planning. By employing multiple scattered beams, clinicians can create a broad and spread-out Bragg peak, optimizing the dose distribution within the target volume. It is essential to note that multiple scattering can also lead to range staggering, which affects the proton beam's range.

The choice of materials used in various components of proton therapy equipment, such as the vacuum chamber, range shifter, and dose monitor, is influenced by their density. Lighter materials, such as aluminum or plastic, are generally preferred to minimize scattering and energy loss. These design choices contribute to the overall efficacy and precision of proton therapy.

The third lecture provided an overview of PSI's technology and compared cyclotrons and synchrotrons in the context of proton therapy. Efficiency and contamination were noted as critical factors in particle accelerators.

The fourth lecture focused on the radiobiology of proton therapy, emphasizing the varying relative biological effectiveness (RBE) and its dependence on multiple factors. Currently, an RBE value of 1.1 is used as a standard. It is important to note that the RBE depends on various factors, including the cell line, organ, endpoint, alpha-beta ratio, recovery, linear energy transfer (LET), dose, dose rate, and fractionation. The concept of gene-directed radiotherapy was also introduced.

Day 1 concluded with clinical discussions and hands-on case practice sessions, focusing on adult central nervous system (CNS) tumors, such as low-grade gliomas, chordomas, and chondrosarcomas (Table 1) [5].

**Table 1:** Chordomas and Chondrosarcomas – PSI experience; N = 222, 1998-2012, Median Age = 43 years, Median Follow up = 57 months

Gender	Male 53%	Female 47%	
Histology	Chordoma 68%	Chondrosarcomas 32%	
Proton Therapy Indication	At Presentation 77%	Recurrence 23%	
Brainstem Compression	No 68%	Yes 11%	Abutment 21%
Optic Pathway Compression	No 69%	Yes 11%	Abutment 20%

Low-grade gliomas represent 46.9% of tumors treated with proton therapy. Studies comparing intensity-modulated radiation therapy (IMRT) using photons to proton therapy demonstrated proton therapy's advantage in reducing the integral dose to normal brain tissue, which correlates with improved quality of life [6].

Chordomas, diagnosed with brachyury, and chondrosarcomas have distinct clinical features and outcomes. Brachyury is a notochordal developmental transcription factor 100% specific for chordomas [7]. Surgery is paramount for chordomas, and irradiating gross tumors is challenging due to their proximity to the brainstem. Clivus chordomas are particularly difficult to treat, with innovative approaches such as 'fat spacers' increasing the distance between the tumor and critical structures, allowing higher radiation doses while minimizing damage to healthy tissues. Intensity Modulated Proton Therapy (IMPT), also known as Multi-Field Optimization, offers increased precision in targeting tumors and is useful for clivus chordomas. An example of typical doses used to treat these tumors is given in Table 2.

**Table 2:** Dose Prescription and Constraints for Chordoma and Chondrosarcomas at Paul Scherrer Institute RBE - Relative Biological Effective Dose

VOLUME/OAR	Definition	Dose
Gross Tumor Volume	Post-op Tumor + high-risk area	74Gy RBE (Chordoma) 70Gy RBE (Chondrosarcoma)
Clinical Tumor Volume	Post-op Tumor bed including the pre-op extension	54Gy RBE
Brainstem		Max 64Gy RBE (Surface) Max 54Gy RBE (Centre)
Spinal Cord		Max 60Gy RBE (Surface) <50.4Gy RBE (Centre)
Optic Chiasm		Max 60Gy RBE
Cochlea		Max 36Gy RBE Mean 30Gy RBE
Hippocampus		40% < 7-3Gy RBE
Temporal Lobe		<20Gy RBE

Ongoing clinical trials investigate the use of vaccines, such as the Brachyury vaccine, in combination with proton therapy [8]. The size of clivus chordomas is a critical determinant of treatment success and radiation-induced toxicity [9]. Smaller tumors allow better local control, while larger tumors increase the risk of complications and necessitate more surgeries.

Spinal tumors present unique challenges in proton therapy. The first IMPT spine treatment was presented by PSI, utilizing a 'donut-shaped' dose distribution [10]. Metal implants, such as screws, should be placed above and below the PTV (Planning Target Volume). Patients are often treated in a prone position to protect the kidneys and small bowel.

In pediatric and adolescent/young adult (AYA) patients, CNS tumors form most cases. Surgery plays a key role in providing better tumor control and survival. Proton therapy craniospinal irradiation necessitates an excellent team and setup challenges in treating pediatric patients, including neutron dose, uncertainties, clinical data, availability, and cost. However, cost analyses have shown that proton therapy is a viable option for pediatric CNS tumors. Some studies have reported higher radiation necrosis in the brain [11].

For diffuse intrinsic pontine glioma (DIPG), proton therapy is not typically employed at PSI due to the aggressive nature of the tumor and limited survival, as it is difficult to justify the use of proton therapy in these cases.

Day 2 of the conference began with lecture nine, which discussed treatment planning for Pencil Beam Scanning and IMPT (Intensity Modulated Proton Therapy). Single Field Uniform Dose (SFUD) planning, also known as Single Field Optimization (SFO), was introduced, followed by an explanation of IMPT and its similarities to IMRT in photon therapy. Distal Edge Tracking (DET) and motion mitigation strategies were also discussed to minimize the effects of uncertainties in proton therapy. DET is an Intensity-Modulated Proton Therapy (IMPT) technique that situates the Bragg peak at the distal boundary of the target volume, facilitating accelerated radiation administration. Through intensity modulation, this technique achieves homogeneous dose deposition within the region of interest [12].

**Figure 6:**
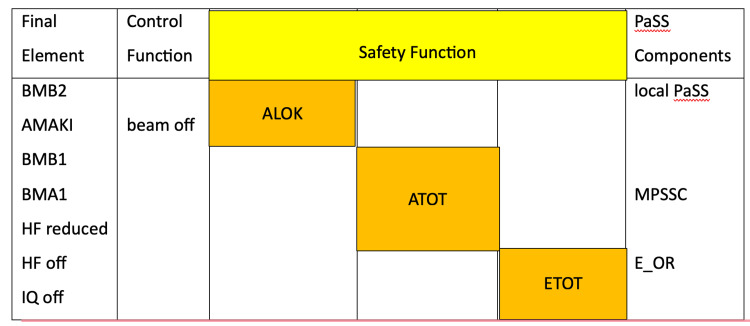
Three levels of Safety Check before delivery of treatment at Paul Scherrer Institute. Reproduced from the lecture on Safety checks PSI WINTERSCHOOL 2018.

**Figure 7:**
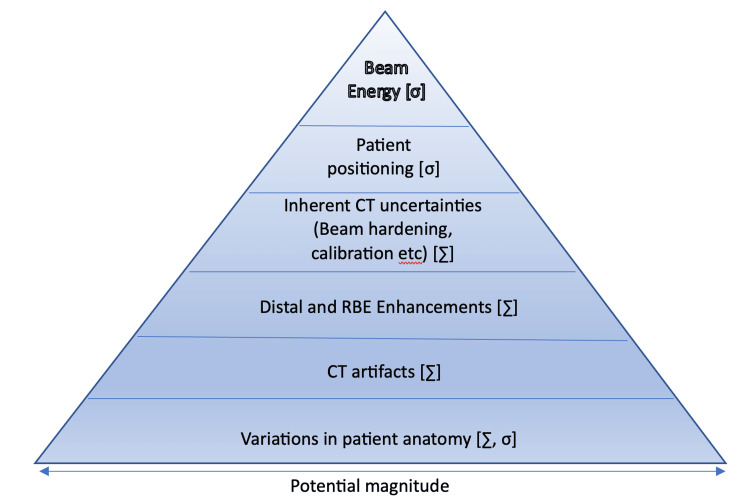
Bermuda Triangle of Uncertainties ; Reproduced with permission from the PSI WINTERSCHOOL 2018 lecture on Uncertainties by Prof. Tony Lomax. The uncertainty and the errors they lead to can either be systematic {∑] or random [σ]. Computed Tomography (CT), Relative Biological Effectiveness (RBE)

Lectures 11 to 14 focused on proton therapy workflow including safety checks (Figure 6), modulating delivery, and controlling delivery, while also focusing on the various uncertainties in proton therapy delivery and ways to overcome them (Figure 7).

Lecture 15 explored the importance of relative biological effectiveness (RBE) in proton therapy. The study on medulloblastoma patients was mentioned, highlighting the significance of LET in proton therapy [13]. Lecture 16 examined the risk of radiation-induced second malignancies, emphasizing the importance of considering absorbed dose, neutron dose, and imaging dose in treatment planning.

Lecture 17 discussed head and neck (H&N) treatments and re-irradiation, emphasizing the importance of careful planning and monitoring in proton therapy for H&N cancers. The final lecture on gastrointestinal (GI) cancers highlighted the benefits of proton therapy for hepatocellular carcinoma (HCC) and cholangiocarcinoma, as well as the importance of considering KRAS and p53 status when treating liver tumors as they may be radioresistant [14,15].

It was interesting to note the various differences between the two Gantries (Probeam`TM Vs Gantry 2) (Table 3).

**Table 3:** Differences between the GANTRY 3 (VARIAN) and GANTRY 2 (PSI). Reproduced from the PSI Winterschool 2018 lecture on Physics by S.Safai

	Varian Probeam	PSI G2
Pencil beams	70-230 MeV, comparable properties	70-230 MeV, comparable properties
Scanning speed	++	+
Layer switching	+	++
Gantry range	360 degrees	180 degrees
Minimum Monitor Units	13-65x lower at G2 => necessity to reduce spot density => number of spots Probeam/G2 = 0.3	13-65x lower at G2 => necessity to reduce spot density => number of spots Probeam/G2 = 0.3
Field size	30x40 cms	11x19 cms
Range shifter	Manual exchange	Automatically driven on a spot basis
Snout	Carries only a range shifter	Carries monitors + range shifter
In-room imaging	kV-kV / CBCT 2D/2D & 3D/3D matching	In-room CT Point-based registration on scouts
Table accuracy	Imaging upon every field!?	Fx based imaging
Couch top supporting rails	Yes	No
Reliability	??	++
Treatment Planning System	Eclipse	PSIPIan
Efficiency	Faster dose calculation, Dicom compliant, Easier adaptation, Better, user friendly	
4D dose calculation	No	Yes, for various rescanning regimes
Logfile calculation	No	Yes
MU calculation accuracy	(+/-2 %)	Requires experimentally determined correction
Lateral spot position correction	Not supported	Experimentally determined correction
Robustness	Evaluation Optimization Fx-based	Evaluation Treatment based

Compact systems, such as synchrocyclotrons, compact cyclotrons, and compact synchrotrons, aim to minimize the size and complexity of proton therapy facilities, making them more accessible and cost-effective. Laser-guided proton acceleration, although challenging, represents a potential future innovation in the field of proton therapy.

Conclusions

The learning curve in proton therapy can be effectively navigated by gradually progressing from simpler, non-mobile tumors to more challenging cases involving tumor motion. Continued education, training, and collaboration with experienced colleagues are essential in this process.

The workshop highlighted that proton therapy has not been as effective in treating prostate malignancies as anticipated. However, exploring alternative planning strategies, such as combining single-field optimized beams with IMPT, could potentially improve treatment outcomes by redistributing dose away from critical structures like the femoral heads. Using a tissue-equivalent bolus, as suggested during the workshop, might offer an alternative to re-absorbers for treating superficial tissues, potentially minimizing scatter, and allowing for precise dose delivery. This approach could be particularly suitable for post-mastectomy chest wall treatments if proven cost-effective.

Finally, the implementation of in-room lasers for daily patient positioning can enhance treatment accuracy and consistency when imaging is not performed regularly. While external surface markers may not perfectly correlate with internal tumor changes, the use of lasers can still contribute to improved treatment positioning.

## References

[1] (2023). PSI Winter School for Proton Therapy 2023. https://indico.psi.ch/event/13263/.

[2] Wilson RR (1946). Radiological use of fast protons. Radiology.

[3] Kardar L, Li Y, Li X (2014). Evaluation and mitigation of the interplay effects of intensity modulated proton therapy for lung cancer in a clinical setting. Pract Radiat Oncol.

[4] Paganetti H, Bortfeld T, Delaney TF (2006). Neutron dose in proton radiation therapy: in regard to Eric J. Hall (Int J Radiat Oncol Biol Phys 2006;65:1-7). Int J Radiat Oncol Biol Phys.

[5] Weber DC, Rutz HP, Pedroni ES (2005). Results of spot-scanning proton radiation therapy for chordoma and chondrosarcoma of the skull base: the Paul Scherrer Institut experience. Int J Radiat Oncol Biol Phys.

[6] Freeman AI, Boyett JM, Glicksman AS, Brecher ML, Leventhal BG, Sinks LF, Holland JF (1997). Intermediate-dose methotrexate versus cranial irradiation in childhood acute lymphoblastic leukemia: a ten-year follow-up. Med Pediatr Oncol.

[7] Miettinen M, Wang Z, Lasota J, Heery C, Schlom J, Palena C (2015). Nuclear Brachyury expression is consistent in chordoma, common in germ cell tumors and small cell carcinomas, and rare in other carcinomas and sarcomas: an immunohistochemical study of 5229 cases. Am J Surg Pathol.

[8] DeMaria PJ, Bilusic M, Park D (2020). A randomized, double-blind, phase II clinical trial of GI-6301 (yeast-brachyury vaccine) versus placebo in combination with standard of care definitive radiotherapy in locally advanced, unresectable, chordoma. J Clin Oncol.

[9] Combs SE, Baumert BG, Bendszus M (2021). ESTRO ACROP guideline for target volume delineation of skull base tumors. Radiother Oncol.

[10] Rutz HP, Lomax AJ (2005). Donut-shaped high-dose configuration for proton beam radiation therapy. Strahlenther Onkol.

[11] Kralik SF, Ho CY, Finke W, Buchsbaum JC, Haskins CP, Shih C-S (2015). Radiation necrosis in pediatric patients with brain tumors treated with proton radiotherapy. AJNR Am J Neuroradiol.

[12] Albertini F, Casiraghi M, Lorentini S, Rombi B, Lomax AJ (2011). Experimental verification of IMPT treatment plans in an anthropomorphic phantom in the presence of delivery uncertainties. Phys Med Biol.

[13] Timmermann B, Lomax AJ, Nobile L (2007). Novel technique of craniospinal axis proton therapy with the spot-scanning system: avoidance of patching multiple fields and optimized ventral dose distribution. Strahlenther Onkol.

[14] Kim JY, Lim YK, Kim TH (2015). Normal liver sparing by proton beam therapy for hepatocellular carcinoma: comparison with helical intensity modulated radiotherapy and volumetric modulated arc therapy. Acta Oncol.

[15] Bush DA, Smith JC, Slater JD (2016). Randomized clinical trial comparing proton beam radiation therapy with transarterial chemoembolization for hepatocellular carcinoma: results of an interim analysis. Int J Radiat Oncol Biol Phys.

